# Influence of Aluminum Surface Treatment on Tensile and Fatigue Behavior of Thermoplastic-Based Hybrid Laminates

**DOI:** 10.3390/ma13143080

**Published:** 2020-07-10

**Authors:** Selim Mrzljak, Maik Trautmann, Guntram Wagner, Frank Walther

**Affiliations:** 1 Department of Materials Test Engineering (WPT), TU Dortmund University, Baroper Str. 303, D-44227 Dortmund, Germany; frank.walther@tu-dortmund.de; 2 Institute of Material Science and Engineering (IWW), Chemnitz University of Technology, Erfenschlager Str. 73, D-09125 Chemnitz, Germany; maik.trautmann@mb.tu-chemnitz.de (M.T.); guntram.wagner@mb.tu-chemnitz.de (G.W.)

**Keywords:** fiber metal laminate, thermoplastic, AA6082, surface treatment, double notch shear test, fatigue behavior, damage mechanisms, instrumented fatigue testing, digital image correlation

## Abstract

Hybrid laminates consist of layers of different materials, which determine the mechanical properties of the laminate itself. Furthermore, the structure and interfacial properties between the layers play a key role regarding the performance under load and therefore need to be investigated in respect to industrial applicability. In this regard, a hybrid laminate comprised of AA6082 aluminum alloy sheets and glass and carbon fiber-reinforced thermoplastic (polyamide 6) is investigated in this study with a focus on the influence of aluminum surface treatment application on tensile and fatigue behavior. Four different aluminum surface treatments are discussed (adhesion promoter, mechanical blasting, phosphating, and anodizing), which were characterized by Laser Scanning Microscopy. After the thermal consolidation of the hybrid laminate under defined pressure, double notch shear tests and tensile tests were performed and correlated to determine the resulting interfacial strength between the aluminum sheet surface and the fiber-reinforced plastic, and its impact on tensile performance. To investigate the performance of the laminate under fatigue load in LCF and HCF regimes, a short-time procedure was applied consisting of resource-efficient instrumented multiple and constant amplitude tests. Digital image correlation, thermography, and hysteresis measurement methods were utilized to gain information about the aluminum surface treatment influence on fatigue damage initiation and development. The results show that fatigue-induced damage initiation, development, and mechanisms differ significantly depending on the applied aluminum surface treatment. The used measurement technologies proved to be suitable for this application and enabled correlations in between, showing that the hybrid laminates damage state, in particular regarding the interfacial bonding of the layers, can be monitored not just through visual recordings of local strain and temperature development, but also through stress-displacement hysteresis analysis.

## 1. Introduction

Hybrid laminates of light metals and fiber-reinforced plastics (FRP), also known as Fiber Metal Laminates (FML), combine the advantageous properties of both material groups in one material compound. Besides a better damage tolerance compared to FRP, the FML have a lower density and higher strength than monolithic metal alloys. These material compounds are of great importance, especially as structural materials in aircraft construction compared to conventional aluminum alloys, due to the lower crack growth based on the fiber bridging effect [1,2,3]. Much of the scientific research especially in the field of mechanical and fatigue properties is based on GLARE, which is a multi-layer combination of aluminum alloy AA2024 and glass fiber-reinforced epoxy resin layers [3,4,5,6,7,8,9]. The elaborate and complex production of the thermosetting-based multi-layer materials is time- and cost-intensive and unsuitable for large-scale production. Hybrid laminates based on fiber-reinforced thermoplastics in combination with aluminum alloys of the 5000 and 6000 series are an interesting alternative. Polypropylene [10], polyamide 6 (PA6) [11], and thermoplastic polyurethane [12] are used as matrix materials in the FRP layer. The layered combination of unidirectional carbon fiber-reinforced PA6 and aluminum sheets AA6082 is known as CAPAAL. The individual layers are joined by a thermal pressing process. The used aluminum alloy AA6082 is precipitation hardenable, which is excellent to anodize, corrosion resistant, and is often used in lightweight mass products such as automobiles. It has been shown that hybrid laminates with a thermoplastic matrix offer basic formability [13,14]. By heating above the melting temperature of the matrix, complex structures can be formed [15,16]. Bending fatigue tests on CAAPAL with mechanically blasted AA6082 sheets revealed at low loads only crack initiation in the aluminum. Fiber cracks and delamination were observed at higher deflections. [17].

An essential aspect of the hybrid laminates is the suitable adhesion between the fiber-reinforced plastic and the aluminum alloy. Surface treatments to the metal sheets such as mechanical treatment, chemical etching, or electrochemical treatment can improve wettability and adhesion between the metal and polymer, impacting on the mechanical properties of metal fiber laminates (e.g., in [18,19,20]). For GLARE laminates, different mechanical (grinding, brushing) and chemical methods for the surface treatment of EN AW-2024-T3 sheets, which were processed subsequently into hybrid laminates GLARE 4/3, were investigated [21]. By grinding with coarse sandpaper (100 grit), the highest interlaminar shear strengths of 72 MPa were achieved for such pretreatments.

The investigation of different surface treatment processes for aluminum AA6082 components in thermoplastic-based hybrid laminates CAPAAL showed that plasma electrolytic anodic oxidation and mechanical blasting lead to less than 20 MPa in a simple lap shear test [22]. One possibility to determine the shear strength is the examination of thermally joined single lap samples, which are easy to produce but do not reflect the real manufacturing process of hybrid laminates. Another test method to measure the interlaminar properties of hybrid laminates with ductile components is the short-beam method according to DIN EN ISO 14130, but this often leads to a failure of the specimens, which does not allow any statement about the interface. Therefore, in this study, a double notched sample in accordance with the ASTM D3846-08 was utilized to measure the shear strength between a surface-treated aluminum alloy and fiber-reinforced polyamide 6. In [23], the method was successfully applied to measure the interlaminar shear properties between surface-treated aluminum alloy sheets and the matrix of glass fiber-reinforced epoxy. Results between 20 and 70 MPa were achieved, and it was found that a nanoscale sculpturing realized by an etching process significantly increases the interlaminar properties.

With regard to the damage behavior of hybrid laminates, there are qualitative analogies between the thermoplastic and thermoset-based variants. The fatigue properties and basic mechanisms of crack propagation in hybrid laminates with thermoset polymer matrices were investigated in detail in the course of the qualification of GLARE [24]. Due to different thermal expansion coefficients of the individual components, internal stresses occur in the laminate during the manufacturing process. These stresses may be released at cut edges or inclusions by delamination and internal cracks, which adversely affect the structural integrity of the material. The degree of residual stress is largely determined by the thermal behavior and mechanical properties of the FRP and the metal as well as by the layered architecture of the FRP and the quality of the internal interfaces of the hybrid laminate [25]. While much research regarding the influence of metal surface treatments on the internal interfaces and quasistatic properties of hybrid laminates is conducted, there is a lack of knowledge about the influence on the cyclic properties, i.e., the fatigue behavior. The aim of this study is the identification of the quasistatic and fatigue performance of thermoplastic-based hybrid laminates with respect to different surface treatments of the metal component. Especially, the metrological-supported investigation of aluminum surface treatment influence regarding damage initiation, development, and mechanisms due to fatigue load is a key aspect of this study. A future use of the materials in e.g., automotive application requires a comprehensive picture of the material behavior, since a long duration of application under fatigue conditions needs to be possible.

## 2. Materials and Methods

### 2.1. Materials and Manufacturing

The investigated hybrid laminate is based on CAPAAL—a layered material compound that consists of surface-treated aluminum alloy sheets AA6082-T4 with a thickness of 0.5 mm and layers of unidirectional fiber-reinforced plastics (FRP). The FRP layer contains multiple layers of CePreg (Cetex Institut gGmbH, Chemnitz, Germany), a continuously produced unidirectional glass (GFR-PA 6), and carbon fiber-reinforced polyamide 6 (CFR-PA 6) with a calculated fiber volume fraction of 0.5. To compensate for the mismatch in the coefficient of thermal expansion between the aluminum alloy and the CFR-PA 6, an additional foil of PA 6 with a thickness of 40 µm and a layer of GFR-PA 6 was added.

**Figure 1:**
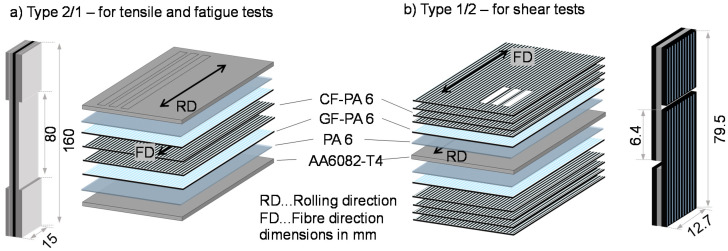
Hybrid laminates composed of aluminum alloy AA6082-T4 and a graded structure of glass and carbon fiber-reinforced polyamide 6 in (**a**) a 2/1-configuration for tensile and fatigue tests and (**b**) a 1/2-configuration to determine the interlaminar shear strength.

The consolidation of the materials with a size of 260 × 260 mm^2^ took place in a dip-edged tool with a laboratory platen press Collin PM300. A maximum temperature of 295 °C was held for 24 min with a pressure of 1.5 MPa. For the tensile tests, a hybrid laminate with 2/1-configuration was used, consisting of two layers of aluminum alloy AA6082 and between these unidirectional FRP (Figure 1a). To investigate the interlaminar shear strength between the metal component and the FRP, the aluminum alloy was positioned in the middle, and the graded FRP layers are arranged on top of both sides. This build-up is referred to as a 1/2-configuration (Figure 1b). After pressing, the samples were cut by a water jet.

### 2.2. Aluminum Surface Treatments

The surface of the aluminum alloy AA6082-T4 was modified using four different treatments:

(I). *Adhesion promoter:* After phosphating (see *III*), the co-polyamide VESTAMELT (Evonik Resource Efficiency GmbH, Marl, Germany) was applied evenly to the sheet as a powder utilizing an electrostatic spray coating and then melted in an oven at 200 °C. This forms a uniform glassy adhesion film with an approximate thickness of 75 µm.
(II). *Mechanical blasting:* The sheets were blasted manually with a pressure of 1 bar on both sides by using high-grade Al_2_O_3_ with a grain size F24 (600–850 µm) in a working distance of 100 mm. The blasting material is applied at an angle of 90° to the surface. The duration for blasting a sheet of 260 × 260 mm^2^ on both sides is 170 s (8 cm^2^/s).
(III). *Phosphating:* The phosphating was carried out using the Bonderite M-ZN process from Henkel. By dipping the sheets in a zinc phosphate solution, a conversion layer of small crystalline zinc-phosphatic mangan will be formed, which typically improves the adhesion of the lacquer.
(IV). *Anodizing:* After a pretreatment by etching the sheets in 10% sodium hydroxide and subsequent pickling, a 15 µm thick layer of Al_2_O_3_ was formed under direct current in sulfuric acid.

### 2.3. Chracterization and Testing Methodes

#### 2.3.1. Aluminum Roughness and Laminate Microstructure

The surface roughness parameters arithmetic mean deviation (S_a_), root mean squared (S_q_), and skewness (S_sk_) of the treated aluminum sheets were determined by a Keyence VK X200 (Keyence Deutschland GmbH, Neu-Isenburg, Germany) laser scanning microscope (LSM) in an area of 290 × 200 µm^2^ with 10 analyzed lines of each sample. After consolidation of the hybrid laminates, the thicknesses were examined on cross-sections by light microscopy and a scanning electron microscope Zeiss LEO 1455VP (Carl Zeiss AG, Oberkochen, Germany).

#### 2.3.2. Quasistatic Properties

**Figure 2:**
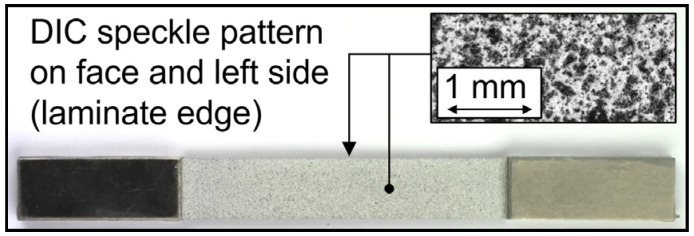
Prepared specimen with end tabs and digital image correlation (DIC) pattern for deformation analysis.

The interlaminar shear strength was measured on a universal testing system Zwick 20 kN on water jet cut samples of the 1/2 configuration with double-notched samples in geometrical accordance with ASTM D3846-08, as shown in Figure 1b. In contrast to the standard, a tensile load was chosen instead of the proposed compression test, as otherwise, the FRP would buckle. Tensile properties were identified through tests on the 2/1 configuration in accordance with DIN EN 527-5 with water-jet cut samples type A (length: 160 mm, width: 15 mm) on a servo-hydraulic testing system (Shimadzu EHF-EV50, max. force F_max_ = ± 50 kN). The gauge length was reduced to 80 mm to gather high-resolution information about local material reactions by the measurement instrumentation of a smaller area. The water jet cut edges were ground with SiC abrasive paper down to P4000 to reduce notches. To reduce stress concentrations at the transitional area of the clamping and testing area, aluminum tabs with a thickness of 1.75 mm were adhesively bonded to the clamping area of the specimen with epoxy (Figure 2). The aluminum tab and clamping area of the specimen were mechanically blasted for adhesion enhancement. A minimum of three samples for each surface treatment was tested.

#### 2.3.3. Cyclic Properties

**Figure 3:**
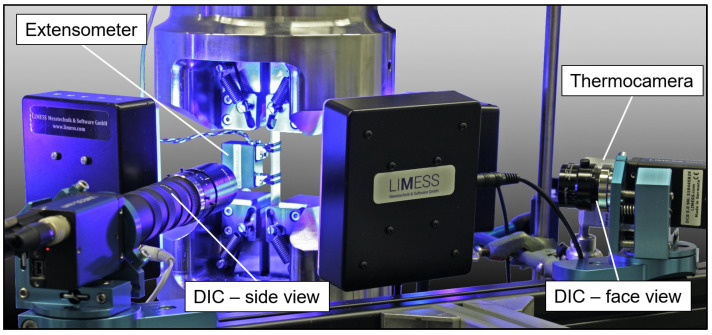
Experimental setup containing a servo-hydraulic testing system, digital image analysis cameras perpendicular to the specimen (face and side view), thermocamera, and tactile extensometer.

For fatigue testing, the same specimen geometry and preparation as for the quasistatic tensile tests were used. A short-time procedure (e.g., in [26]) was used for fatigue investigations, combining multiple amplitude tests (MAT) and constant amplitude tests (CAT), which were carried out on a servo-hydraulic testing system (EHF-EV50 from Shimadzu Europe GmbH, Duisburg, Germany, maximum force F_max_ = ± 50 kN, Figure 3) with sinusoidal tension-tension load-time function at a stress ratio R = 0.1 and testing frequency f = 10 Hz under ambient temperature with regard to ISO 13003. For the MAT, the maximum starting stress σ_max,start_ was 50 MPa, followed by a stepwise stress increase of Δσ_max_ = 25 MPa per ΔN = 10^3^ cycles up to specimen failure. The CAT stress levels were derived from the material reactions in MAT, between the onset of first microstructural change (high cycle fatigue (HCF)) and specimen failure (low cycle fatigue (LCF)).

The changes in the microstructures and properties of specimens were measured by a combination of systems to enable a combined investigation of multiple measurands (e.g., in [27]). A Limess Q400 digital image correlation (DIC) system (Limess Messtechnik und Software GmbH, Krefeld, Germany) was used for surface deformation analysis of the front aluminum sheet (precision lens, 28 mm focal length) and laminate edge (macro lens, 70 mm focal length, with extension tubes) to observe interface reactions between the laminate partners (DIC speckle pattern and area analysis directions are shown in Figure 2 and Figure 3). DIC pictures were taken at maximum stress with the help of a Limess Maxtrigger box. A MicroEpsilon TIM 160 thermocamera (Micro-Epsilon Messtechnik GmbH & Co. KG, Ortenburg, Germany) recorded the front aluminum surface temperature. For the investigation of stress-strain hysteresis development, a Shimadzu TCK 1 LH (l_0_ = 25 mm, Δl = ± 1 mm) extensometer, and the engineering stress, derived from the load cell at the initial specimen area, were used. Additionally, the machine piston displacement (s) was recorded by a linear variable differential transformer (LVDT) for stress-displacement hysteresis analysis, as the occurring development of aluminum cracks and aluminum delamination from the FRP led to a slippage of the extensometer, resulting in recorded data deviations and less reliability.

## 3. Results and Discussion

### 3.1. Aluminum Roughness and Laminate Microstructure

**Figure 4:**
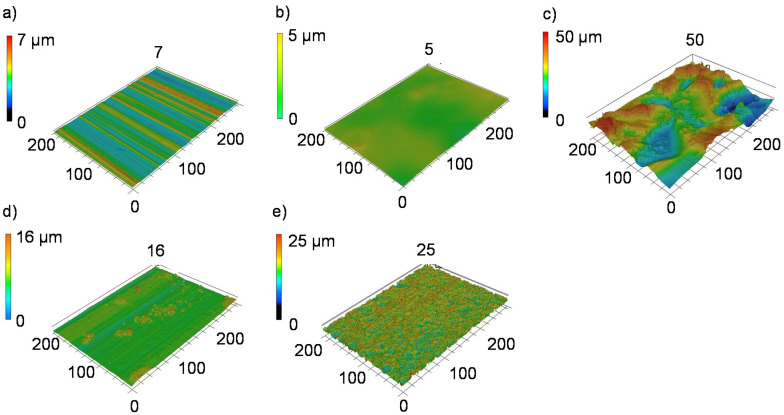
Laser scanning microscopy of (**a**) untreated and surface-treated aluminum alloy AA6082: (**b**) adhesion promoted, (**c**) mechanical blasted, (**d**) phosphated, and (**e**) anodized.

The quantification of the surface roughness parameters measured by laser scanning microscopy (LSM, Figure 4) shows that the mechanical blasting and anodizing process, in particular, can cause an increase of S_a_ and S_q_ compared to an untreated aluminum sheet, whereas the other investigated processes do not cause any pronounced changes, as shown in Table 1. The increase in roughness is associated with an increase in the total surface area and a formation of further bonding areas for the thermoplastic melt. The skewness describes the distribution of the height profile to the reference line. A positive value, as measured by phosphating, describes a profile determined by narrow peaks, which is caused by the grown phosphate crystals. In contrast, the anodized surface is characterized by a slightly negative skewness.

**Figure 5:**
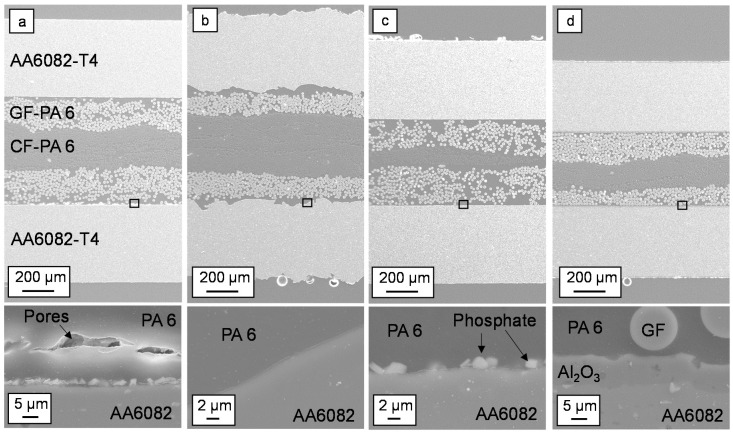
Scanning electron microscope cross-sections of hybrid laminates with an (**a**) adhesion promoted, (**b**) mechanically blasted, (**c**) phosphated, and (**d**) anodized aluminum alloy AA6082-T4 and a graded structure of glass and carbon fiber-reinforced polyamide 6 (PA 6) in a 2/1 configuration, below: detailed area of the interface.

The cross-sections of hybrid laminates show a different material thickness by using identical pressing parameters, as shown in Figure 5. The rough surface of the blasted sheets leads to laminates with the highest thickness. In contrast, the hybrid laminates with the anodized surfaces have a significantly lower thickness, although the arithmetic mean roughness is higher than by phosphating, etching, and adhesion promoter. In particular, the thickness of the CFR-PA 6 layer is significantly less compared to the GFR-PA 6 layer, which indicates that these layers are more strongly displaced from the laminate than the GFR-PA 6 layers. The rheological behavior of the thermoplastic melt during the consolidation process is influenced by the surface treatment, the fibers inside the FRP layer, and the surface material. The results show that the anodization causes the strongest displacement of the thermoplastic material in the process. The material is pressed out through a gap between the dipping edge and tool cover plate. This also results in a strong change in the metal volume content, which in turn influences the strength of the material compounds.

Detailed visual observations of the interface show a connection of the PA 6 without delamination for all variants, as shown in Figure 5a–d. Especially with the mechanically blasted aluminum sheet, a good mechanical interlocking can be achieved by undercut areas, as shown in Figure 5b. Only in the interface with the adhesion promoter, a pore seam between the GFR-PA6 and the adhesion promoter is visible, as shown in Figure 5a. Surfaces with a low surface roughness S_a_ (1 µm smaller than the untreated rolling surface) also show good adhesion to the PA 6. This implies that not only the microscopic form closure but also weak bonding forces between metal and FRP is responsible for the connection.

The cross-sections show the unsteady position of the CFR-PA 6 layer, e.g., Figure 5d. By using the unidirectional orientation of all FRP tapes, small differences in the fiber distribution of the CePreg material lead to displacement processes among each other in the viscous state during the pressing.

### 3.2. Quasistatic Properties

**Figure 6:**
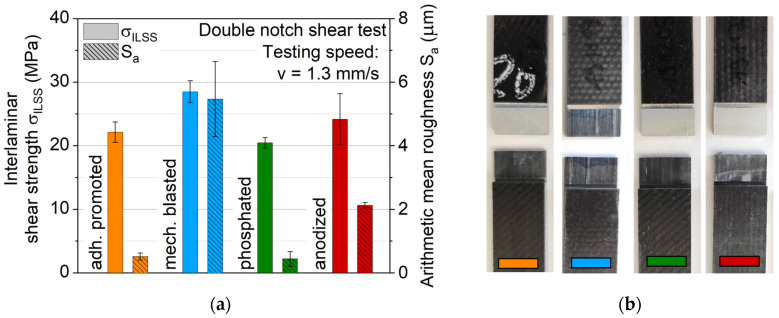
Results of (**a**) an interlaminar shear test of hybrid laminates with surface-treated aluminum alloy AA6082-T4 and a graded structure of glass and carbon fiber-reinforced PA 6 in a 1/2 configuration; (**b**) fractography images.

The results of the interlaminar shear tests are displayed in Figure 6 in comparison to the measured arithmetic mean surface roughness. With a maximum value of 28 MPa for mechanical blasting, a significantly higher value can be determined than those measured on overlap joints of the same material combinations in [22]. On blasted specimens, residues of the FRP can be seen on the surface, which is due to the cohesive failure of the composite material. The real interfacial strength of this surface treatment is even higher than the value determined. It is not possible to draw conclusions about the interfacial strength only from the roughness. The samples with anodized surfaces have similar high shear strength values despite their lower roughness. It is assumed that different adhesion mechanisms are effective. Through mechanical blasting, the PA 6 mechanically interlocks with the surface, whereas with anodization, binding forces are responsible for cohesion.

**Figure 7:**
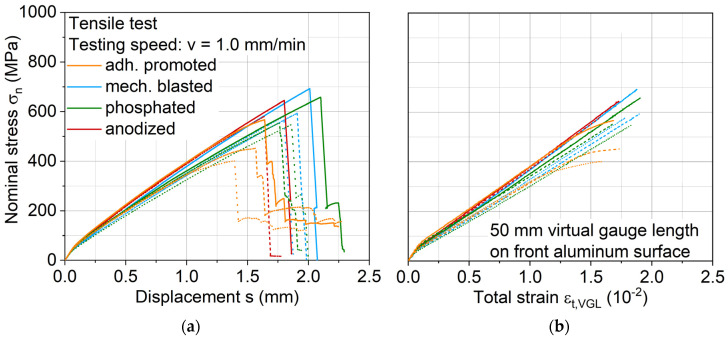
(**a**) Stress-displacement curves and (**b**) stress-strain curves of hybrid laminates with surface-treated aluminum alloy AA6082-T4 and a graded structure of glass and carbon fiber-reinforced PA 6 in a 2/1 configuration. The curve color represents an aluminum surface treatment variant of the hybrid laminate; dotted lines represent different specimens.

Stress-displacement and -strain (measured via a DIC virtual gauge length) curves are displayed in Figure 7, where similar stress-displacement development is found across the investigated aluminum surface treatments, except for adhesion promotion, which shows lower tensile strength and a significant stiffness decrease before failure. DIC images proved that the latter is due to interface failure. At the start of each tensile test, a kink is visible, which is assumed to be representing the exceeding of the aluminum yield stress, resulting subsequently in an elastoplastic deformation of the aluminum and viscoelastic deformation of the FRP.

**Figure 8:**
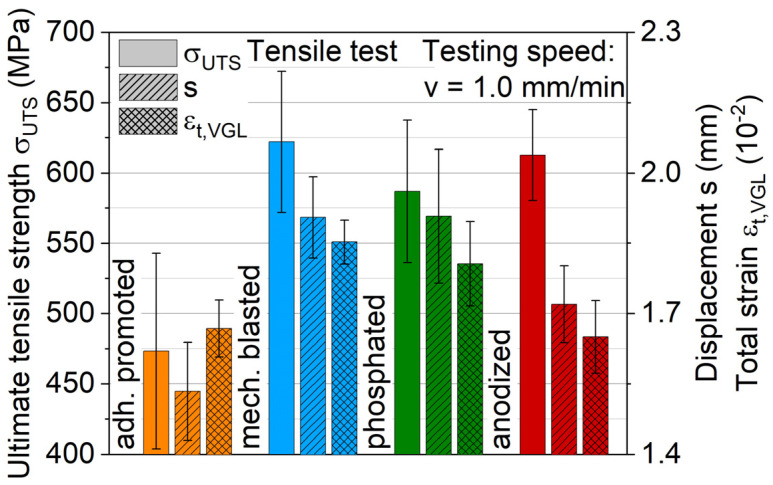
Arithmetic tensile strength and strain values for hybrid laminates with surface-treated aluminum alloy AA6082-T4 and a graded structure of glass and carbon fiber-reinforced PA 6 in a 2/1 configuration.

Measured ultimate tensile strengths and total strains of the hybrid laminates with different aluminum surface treatments are shown in Figure 8. The qualitative results agree with the distribution observed by the interlaminar shear test. Mechanical blasting leads with a maximum average value of 625 MPa. Anodizing shows an ultimate tensile strength of around 610 MPa, while phosphating reaches 580 MPa. No significant differences in strain distribution could be found via DIC analysis of the laminate interface across these three different surface-treated aluminum variants. While these three aluminum surface treatments lead to similar tensile performance, the adhesion promoter achieves significantly lower values of 475 MPa, which can be attributed to extensive delamination of the aluminum leading to an earlier failure of the hybrid laminate. The partially large scattering of the results is due to the inhomogeneity of the fiber distribution inside the FRP component. The displacement process during production causes fiber shifts, which result in different proportions of carbon and glass fibers in the individual specimen.

### 3.3. Cyclic Properties

**Figure 9:**
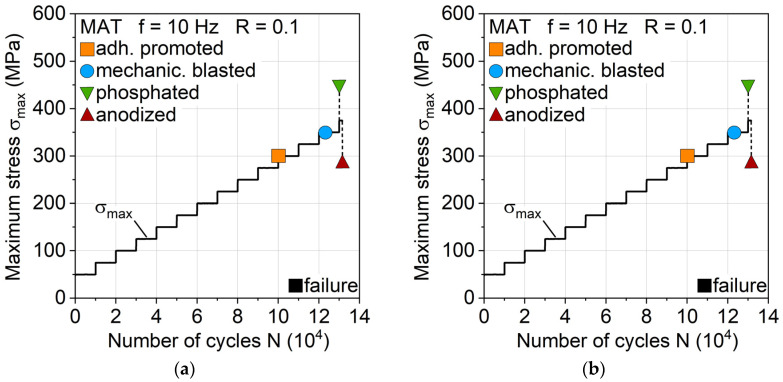
Influence of aluminum surface treatment on (**a**) fatigue strength and life and (**b**) change in temperature during multiple amplitude tests.

For a first estimation of the aluminum surface treatment influence on fatigue behavior, MAT were carried out, which revealed discernible differences with regard to fatigue life and damage development. Anodizing leads to the highest MAT fatigue life, while adhesion promotion treatment shows the lowest (Figure 9a). The change in temperature ΔT reveals differences between the surface treatments, hinting at differing laminate properties and damage progressions (Figure 9b). Mechanical blasting and anodizing show a higher temperature increase earlier than adhesion promotion and phosphating.

**Figure 10:**
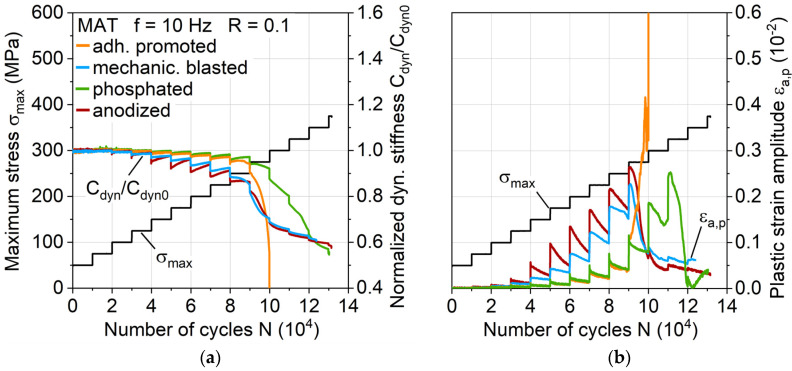
Influence of aluminum surface treatment on (**a**) normalized dynamic stiffness and (**b**) plastic strain amplitude during multiple amplitude tests.

The changes in dynamic stiffness C_dyn_ (change of stress divided through the change of piston displacement: (σ_max_ – σ_min_)/(s_max_ – s_min_), see Figure 10a) illustrate that the earlier temperature increases can be attributed to a load-increased cyclic softening and the hardening processes of the aluminum, which are particularly present in anodized surface treatment. Adhesion promotion and phosphating exhibit the same but delayed cyclic hardening processes, which could be due to the phosphating of both. The plastic strain amplitude ε_a,p_ (Figure 10b) correlates directly to the stiffness and temperature change, revealing the plastically deforming aluminum component as the main cause for the temperature increase. After the temperature maxima, stiffness decrease due to damage propagation in the interface and the fiber-reinforced polymers is visible.

**Figure 11:**
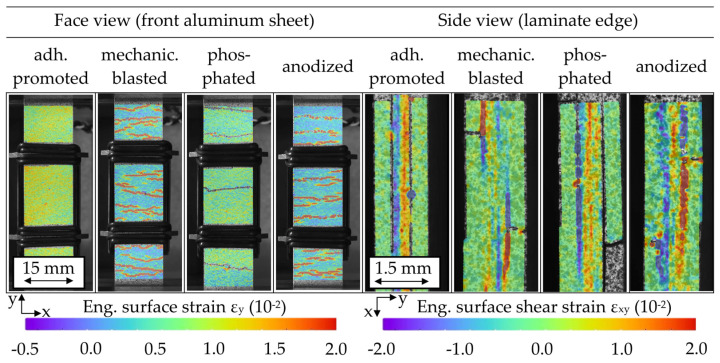
Digital image correlation-based strain analysis images of front aluminum perspective and in-plane laminate perspective immediately before multiple amplitude tests (MAT) failure for hybrid laminates with different aluminum surface treatments.

Through front observation and in-plane observation of the laminar damage propagation via DIC, differences depending on the surface treatment and related cyclic hardening progression were recognized. Figure 11 visualizes the specimen damage through strain analysis, representing the condition immediately before MAT failure. Visible aluminum cracks formed primarily from the outer specimen edges to the inside, developing into interface cracks and delamination. For the variants of later hardening (adhesion promotion and phosphating treatment), substantial amounts of delamination between the aluminum and FRP are present, while the variants that are inclined to an earlier and high quantity of crack initiation (mechanical blasting and anodizing treatment) enable more local reductions of stress concentrations and show therefore locally restricted delamination.

**Figure 12:**
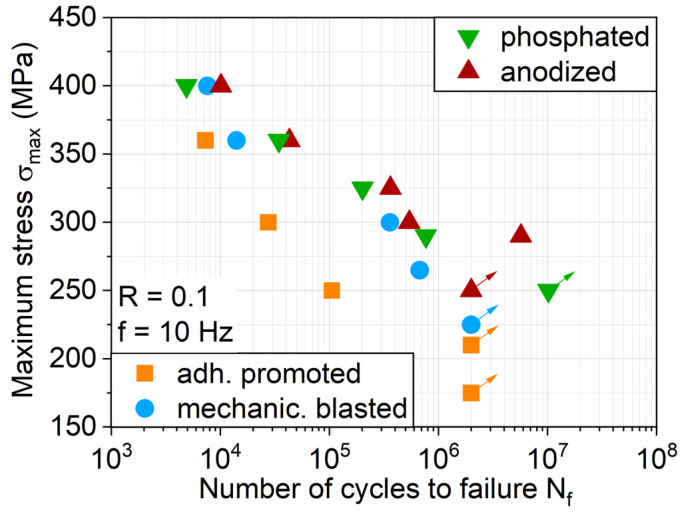
S-N relationship for hybrid laminates with different aluminum surface treatments.

The S-N relationship for each hybrid laminate with the individual treated aluminum surface confirms the MAT findings regarding fatigue lifetime, showing that it is anodized and phosphated in general with the highest fatigue life (Figure 12). The extensive delamination visible for an adhesion promoter during MAT (Figure 11) also takes place in CAT, representing the low interfacial load capacity between metal and the FRP, reducing the fatigue lifetime. DIC analysis during MAT and CAT showed that the phosphated variant delaminates due to fewer cracks to a larger extent, but more gradually than adhesion promotion, leading to similar damage-induced stress relief and distribution as for the anodized and mechanically blasted laminates, as well as fatigue life.

**Figure 13:**
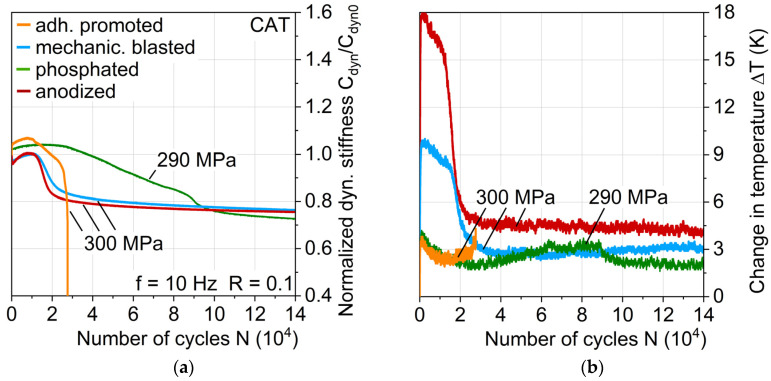
Influence of aluminum surface treatment on (**a**) normalized dynamic stiffness and (**b**) change in temperature (plotted linearly) during constant amplitude tests.

This more gradual damage propagation of the phosphated variant is shown with regard to the stiffness development in CAT exemplary for a maximum stress level of 300 MPa up to 14E4 cycles (which represents damage development up until nearly full aluminum crack extension) in Figure 13a directly compared to the other aluminum surface treatments. The maximum stress level of the shown phosphated variant specimen is 10 MPa lower due to achieving a more evenly distributed impression of the S-N relationship, and in view of the results obtained with regard to fatigue lifetime and behavior, it is considered negligible and therefore comparable to 300 MPa. As was the case for MAT, the cyclic softening and hardening appear for mechanically blasted and anodized laminates, while adhesion promoted and phosphated exhibit cyclic hardening from the beginning of the test.

The change in temperature in Figure 13b also shows the same characteristics as in the MAT, where it directly correlates with the plastic strain amplitude. At this point, it can be seen as established that through MAT, many characteristics of the hybrid laminates behavior appearing in CAT can be derived. Adhesion promoted and phosphated show just small increases in temperature and plastic strain amplitude with an afterward approximately constant leveling, which can be a reason for the postponed and slower damage development compared to the laminates with mechanically blasted and anodized aluminum. This could be attributed to an overall higher stiffness of the phosphated aluminum, leading to a more crack-dominated stiffness decrease rather than a plastic strain-induced stiffness.

**Figure 14:**
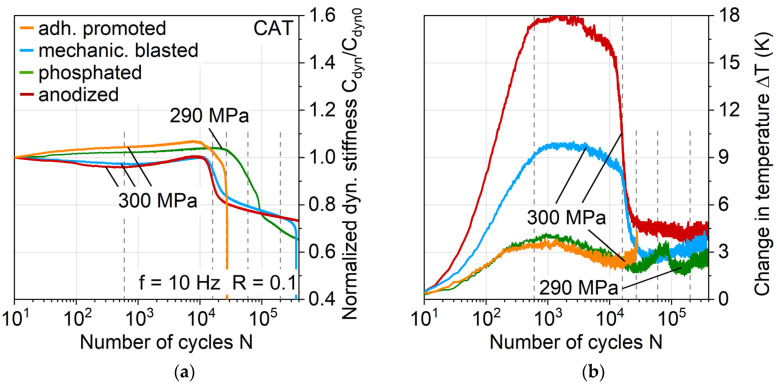
Influence of aluminum surface treatment on (**a**) normalized dynamic stiffness and (**b**) change in temperature (plotted logarithmically) during constant amplitude tests; dotted indicator lines for cycles of hysteresis investigation.

The logarithmic plot of the normalized stiffness (Figure 14a) and change in temperature (Figure 14b) enable a supplementary view of the damage progression by focusing on the major events at the beginning of fatigue load appliance and showing the later on development, which here is up to 4 × 10^5^ cycles. It becomes apparent that the mechanically blasted and anodized laminates undergo a stiffness reduction, inflicting an increase in temperature up to approximately 600 cycles followed by a stiffness increase and starting damage initiation at the maximum stiffness at around 10^4^ cycles. The adhesion promoted and phosphated laminate increased in stiffness up until damage initiation at around 9 × 10^3^ and 2.7 × 10^4^ respectively while showing a significantly lower increase in temperature.

Comparing the initiation time of the large decreases in stiffness with the large decreases in change in temperature, a delay in between those is noticeable. Especially for the mechanically blasted and anodized laminates, an earlier onset of stiffness decrease is visible compared to temperature decrease. Furthermore, the stiffness development of the phosphated variant shows discernable differences: a prolonged decrease in stiffness, and after aluminum crack extension, the normalized dynamic stiffness is reduced more than compared to the mechanically blasted and anodized laminates.

**Figure 15:**
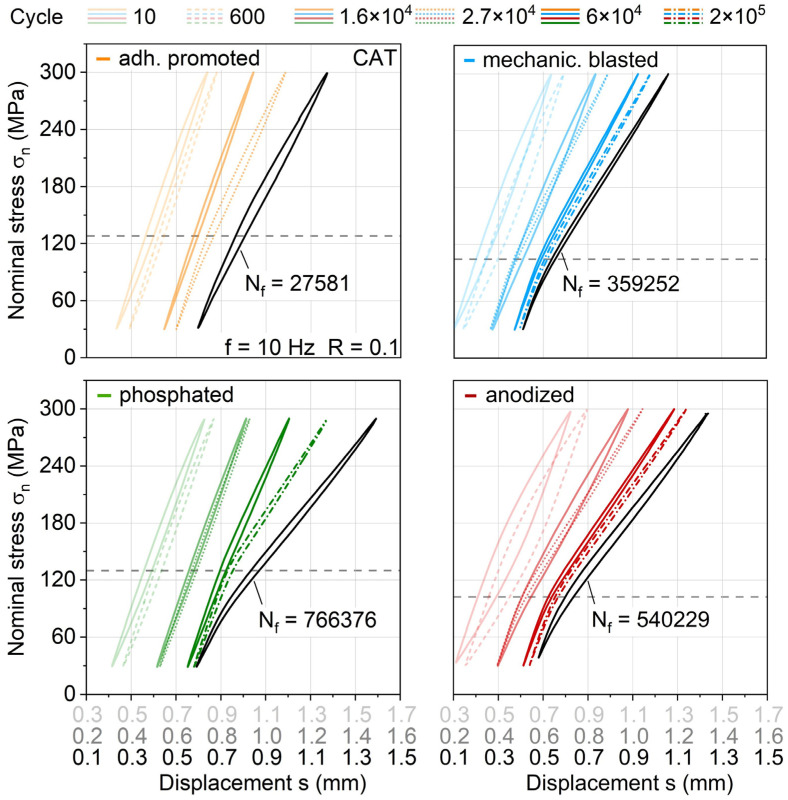
Stress-displacement-hysteresis development in constant amplitude tests with a maximum stress of 300 MPa with regard to aluminum surface treatment; dotted indicator lines visualize maximum stress at which hysteresis starts exhibiting a kink due to aluminum failure.

To get a better understanding of this stiffness and temperature development, the corresponding stress-displacement hystereses are investigated (Figure 15). Single hysteresis curves are plotted for the state right after the fatigue test start (N = 10) and selected numbers of cycles according to the dotted lines in Figure 14 for a direct correlation to the measured stiffness and temperature (and therefore plastic strain amplitude) development. In order to avoid overlapping hystereses and thus reduced visibility, the hystereses are provided with offset x-axes related to an increasing number of cycles, which is indicated by the different saturation of hystereses curves and x-axes print. Looking at the anodized materials, immediately, the decrease in stiffness and high increase in temperature at the beginning of the CAT (Figure 14, N = 10 and 600) can be correlated with a high opening and area of the hysteresis curve, which represents the amount of dissipated energy. Since the other aluminum surface treatment variants show this to a smaller extent, it is assumed that different surface treatment-induced aluminum states regarding mechanical properties are present, which needs to be investigated in further studies through hardness testing, etc. Even if anodized shows the shortest time until stiffness decreases and the highest extent of experienced loss energy, this seems to have no significant impact on the overall lifetime of the hybrid laminate (Figure 12, compare lifetime at a maximum stress of around 300 MPa). However, it immensely affects the stiffness development and therefore duration of maintaining the structural integrity of the laminate (compare, e.g., phosphated).

The most prominent conspicuousness between the different aluminum surface treatments lies in the differing establishment and development of a kink in the hysteresis. Due to the hysteresis development before and after the significant stiffness reduction, it is assumed that the lower part of the hysteresis below the kink represents the full hybrid laminate stiffness, whereas the upper part represents the FRP stiffness with aluminum cracks opened. For all variants, the initial stiffness at load application (full hybrid laminate) stays more or less the same until failure, while the stiffness at load removal (FRP with aluminum cracks open) and the position of the kink point (determined via tangents between loading and unloading curve of the hysteresis; kink point height indicated by dotted lines associated to maximum stress) changes significantly. Moreover, approaching failure, the kink point is lowered in terms of its appearance at a maximum stress level, which is especially visible for the phosphated variant. The stiffness reduction of the phosphated variant, which overall is slower, but higher than for the other treatments, can be traced back to a drastic reduction in the stiffness of the FRP as well as lowering of the kink point. Even though the phosphated variant shows the highest stiffness decrease overall, compared to the anodized and mechanically blasted variant, a comparable fatigue life and kink point height at failure is present. This indicates that the kink point height could be used in further studies as a parameter representative for interface condition and failure prediction.

**Figure 16:**
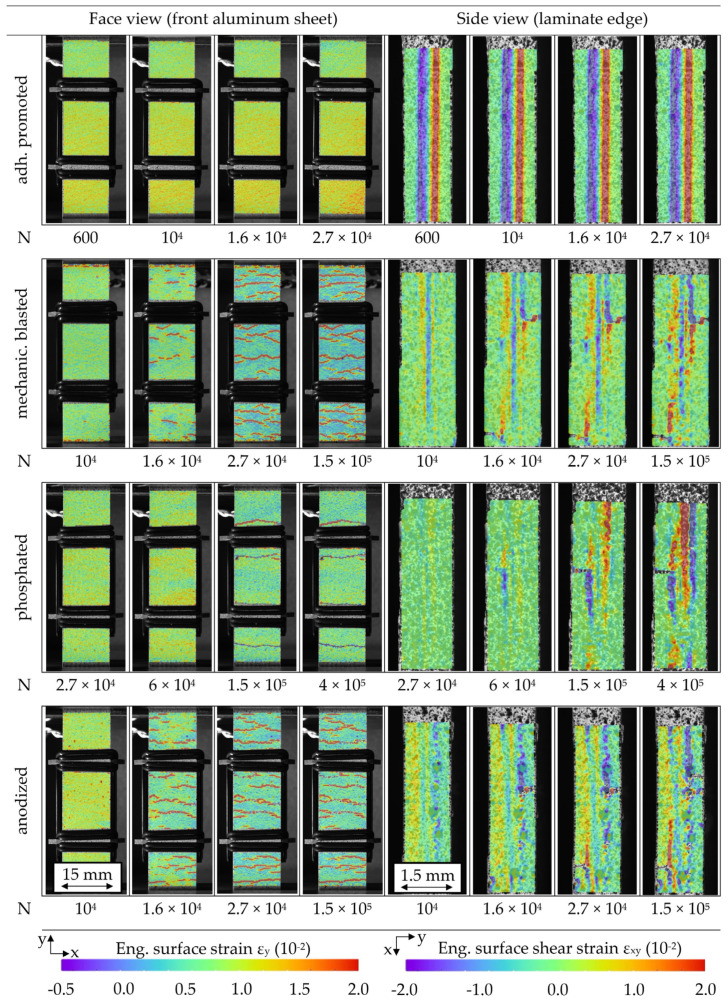
Digital image correlation-based strain analysis images of the front aluminum perspective and in-plane laminate perspective at defined constant amplitude test cycles (σ_max_ = 300 MPa, phosphated 290 MPa) for hybrid laminates with different aluminum surface treatments.

The occurrence of the kink point seems to be related to the failure of the weaker laminate partner aluminum and the height of the kink point to the grade of damage development in the interface to the FRP. Figure 16 shows DIC analysis images for selected cycles representing characteristic states of damage for each aluminum surface treatment variant at applied maximum stress, which support this relationship. While adhesion promoted undergoes extensive interface damage already from the start of the test and fails early due to it, the other three aluminum surface treatments show a gradual damage development. This includes (1) aluminum crack initiation, (2) interface damage initiation, and (3) interface delamination, which can be reflected on the overall stiffness development and also kink point establishment and development.

By combining the results from Figure 14, Figure 15 and Figure 16, for, e.g., mechanical blasting, it can be concluded that the decrease in stiffness visible starts right after 10^4^ with (1) aluminum crack initiation, which is followed by (2) damage initiation in the interface in form of continuing cracks and (3) delamination along the aluminum (see Figure 16, exemplary 1.6 × 10^4^). Taking the aforementioned decrease in temperature into account (Figure 14), it becomes evident that the change in temperature, respectively in relation to plastic strain amplitude, is reduced greatly after the first crack initiation when cracks propagate, not simultaneously. This crack propagation results in the kink of the hysteresis, as shown in Figure 15 exemplary for 2.7 × 10^4^ cycles. With ongoing cycles, the delamination develops further (see Figure 16, exemplary 1.5 × 10^5^), correlating primarily to FRP stiffness decrease. After 1.5 × 10^5^ cycles along with the FRP stiffness decrease, a lowering of the kink point occurs (compare cycle 2 × 10^5^ from Figure 15) while approaching failure. When evaluating the phosphated variant, a different damage development is present. Cracks develop to a smaller extent while having a similar stiffness decrease (compare 6 × 10^4^ from Figure 14 and Figure 16), which is assumed to be due to the higher surface treatment-induced aluminum stiffness, causing more stress concentration relief through interface damage rather than aluminum crack initiation. This seems plausible looking at the smaller number of cracks at 1.5 × 10^5^ (see Figure 16) cycles. Comparing the kink point height in the hystereses at cycle 2 × 10^5^ and N_f_ (Figure 15) with the delamination extension visible in Figure 16 between the exemplary images shown for cycles 1.5 × 10^5^ and 4 × 10^5^, a connection between the reduction of kink point height and interface damage can be seen.

Anodized undergoes a similar fatigue damage development as mechanical blasting, showing differences just in a slightly earlier onset of cracks and more delamination growth, which is attributed to inferior polymer adhesion due to lower surface roughness. Still, as the phosphated variant did, the anodized variant as well shows an overall longer fatigue life than the mechanically blasted, pointing out that the stress concentration relief through delamination expansion does not necessarily appear to have a negative effect on fatigue life, rather a prolonging one.

**Figure 17:**
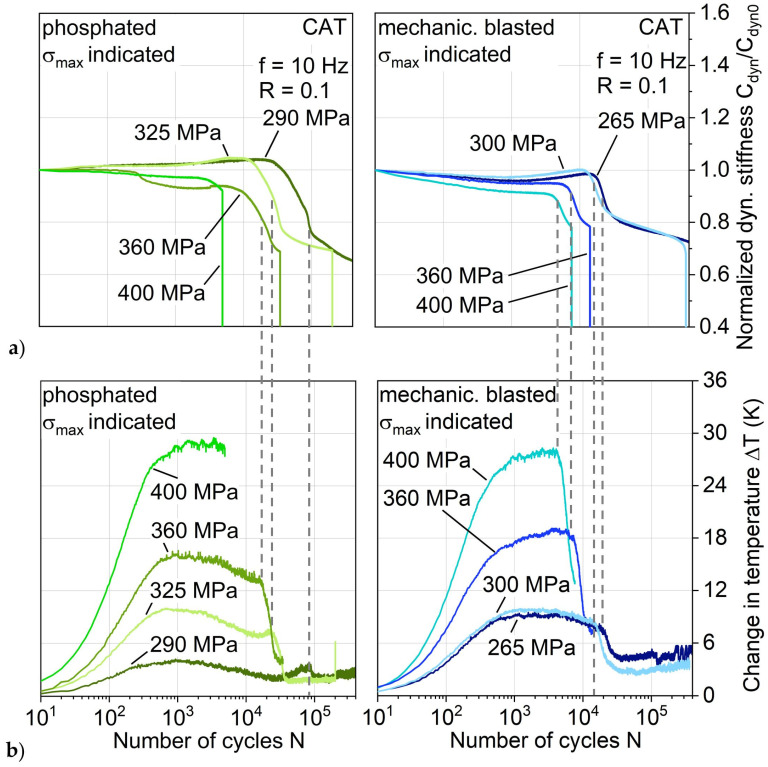
Influence of phosphating and mechanical blasting aluminum surface treatment on (**a**) normalized dynamic stiffness and (**b**) change in temperature (plotted logarithmically) during constant amplitude tests at a variety of σ_max._

In addition to a comprehensive analysis of all the investigated hybrid laminates with different aluminum surface treatments at an exemplary maximum stress level, the phosphated and mechanically blasted variants were compared at different maximum stress levels (Figure 17) to achieve a more profound understanding about the fatigue performance differences. For each of the shown maximum stress levels, a major temperature decrease only occurs after the stiffness has already decreased (see gray dotted lines), not simultaneously with it. This decrease in temperature always seems to occur at similar stiffness losses after the initiation of the strong stiffness decrease, indicating the visible appearance of a hysteresis kink. It can be assumed that the decrease in temperature for each of the maximum stresses shown correlates with the initiation of crack propagation, resulting in the fact that the aluminum can only bear a load as long as the interfaces can transfer it from the FRP to the aluminum. Looking at the phosphated variant, it becomes apparent again that crack initiation is delayed and occurs on a smaller scale, which is why crack propagation only occurs at a significantly higher decrease in stiffness. On the other hand, the phosphated variant has generally a higher overall decrease in stiffness than the compared mechanically blasted variant, which is why, depending on the application, the phosphated variant can be more suitable (longer retention of stiffness with low crack formation) or less suitable (greater delamination propagation with strong stiffness reduction).

## 4. Conclusions

The following conclusions can be drawn from the investigations on the influence of surface-treated aluminum sheets AA6082-T4 in hybrid laminates, based on glass and carbon fiber-reinforced polyamide 6, regarding mechanical properties.

The surface of the aluminum alloy affects the rheological behavior of the melt during the consolidation process. The thickness of the laminates varies with different surface treatments in a pressure-controlled process. Mechanically blasting results in the highest surface roughness and achieves the highest interlaminar shear strength of 30 MPa of all the investigated aluminum surface treatments. However, the ultimate tensile strength of the hybrid laminate does not seem to be heavily dependent on the aluminum surface treatment as long as no delamination occurs as with the applied adhesion promoter, since the mechanically blasted, phosphated, and anodized variants show similar results.

By using multiple amplitude tests, digital image correlation analysis, and hysteresis-stress-strain measurement, an overview about the fatigue performance was established and validated successfully through constant amplitude tests. The fatigue damage development and fatigue life of hot-pressure consolidated thermoplastic-based hybrid laminates are highly influenced by the aluminum surface treatment. Anodizing and phosphating achieve the highest fatigue life, while in comparison, the adhesion promoter led to a significantly reduced fatigue life due to a low interfacial load capacity. Furthermore, results showed that the aluminum surface treatment affects the stiffness development and therefore the duration of maintaining the structural integrity of the hybrid laminate more than the fatigue lifetime itself. It appears that of all the investigated treatments, phosphating enables the longest retention of stiffness with low crack formation but causes greater delamination propagation with the highest overall stiffness reduction, which needs to be considered regarding applications. In conclusion, a graded crack initiation and propagation—which is influenced by the aluminum state, aluminum surface, metal-FRP interface, and the onset of aluminum cyclic hardening—contributes to higher fatigue life, but it needs further investigation regarding the relevant factors for remaining fatigue lifetime after aluminum crack initiation (when mainly the FRP is bearing the load).

Through stress-displacement hysteresis analysis, an aluminum surface treatment-dependent appearance of a hysteresis kink was detected, which seems to be related to aluminum and aluminum-FRP interface crack initiation and propagation. The visible appearance of the hysteresis kink was found to establish at a significant aluminum surface temperature (or correlating plastic strain amplitude) reduction, independent of the applied maximum stress (as long as aluminum cracks initiate). In addition, the position of the kink point showed dependency of the aluminum surface treatment with regard to load-bearing capabilities at applied stress and the grade of damage in the aluminum-FRP interface (mainly delamination). A connection between the kink point height regarding its occurrence at maximum stress and the amount of interface damage can be seen, which needs to be validated in further studies through, e.g., digital image correlation analysis of whole cycles rather than strain states at applied maximum stress.

The interfacial, microstructural differences between the metal and FRP of the hybrid laminate need to be characterized further regarding occurring damage mechanisms for a better understanding of the surface treatments regarding improvement possibilities and should be analyzed during fatigue tests through, e.g., accompanied microscopy. Since the investigated aluminum surface treatments lead to different laminate thicknesses, the related ratio of fiber-reinforced polymer and fiber density needs to be considered for each laminate variant with regard to a relative comparison of the individual and integral laminate partners fatigue performance for a more accurate description of the fatigue performance.

## Figures and Tables

**Table 1:** Roughness parameters of surface-treated aluminum alloy AA6082-T4 measured by laser scanning microscopy transvers to rolling direction.

**Surface Treatment** **AA6082-T4**	**Arithmetic Mean** **S_a_ in µm**	**Root Mean Squared** **S_q_ in µm**	**Skewness** **S_sk_**	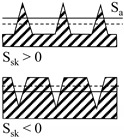
Untreated	0.55 ± 0.12	0.66 ± 0.01	0.6 ± 0.1	
Adhesion promoted	0.49 ± 0.16	0.59 ± 0.18	0.5 ± 0.5	
Mechanically blasted	5.74 ± 1.18	6.88 ± 1.13	−0.2 ± 0.4	
Phosphated	0.67 ± 0.15	0.95 ± 0.24	1.3 ± 0.8	
Anodized	2.13 ± 0.13	2.39 ± 0.10	−0.4 ± 0.1	
